# Home environment factors associated with child BMI changes during COVID-19 pandemic

**DOI:** 10.1186/s12966-024-01634-2

**Published:** 2024-08-02

**Authors:** Carolyn F. McCabe, G. Craig Wood, Gregory J. Welk, Adam Cook, Jennifer Franceschelli-Hosterman, Lisa Bailey-Davis

**Affiliations:** 1 Population Health Sciences, Center for Obesity and Metabolic Research, Geisinger, 100 N. Academy Ave, Danville, PA 17822 USA; 2 Center for Obesity and Metabolic Research, Geisinger, 100 N. Academy Ave, Danville, PA 17822 USA; 3 Department of Kinesiology, University of Iowa, 235 Forker Building, 534 Wallace Road Ames, Iowa City, IA 50011-4008 USA; 4 Department of Nutrition and Weight Management, Geisinger, 100 N Academy Ave, Danville, PA 17822 USA

**Keywords:** Pediatric obesity, Childhood overweight, Family nutrition and physical activity (FNPA), Electronic health record, Retrospective study

## Abstract

**Background:**

The influence of home obesogenic environments, as assessed by the validated Family Nutrition and Physical Activity (FNPA) tool, and child obesity during the COVID pandemic were evaluated using electronic health records in this retrospective cohort study.

**Methods:**

Historical data on BMI and the FNPA screening tool were obtained from annual well-child visits within the Geisinger Health System. The study examined youth ages 2–17 that had a BMI record and an FNPA assessment prior to the pandemic (BMI 3/1/19–2/29/20), 1 BMI record 3 months into the pandemic (6/1/20–12/31/20) and 1 BMI in the second year of the pandemic (1/1/21–12/31/21). Tertiles of obesity risk by FNPA score were examined. Mixed-effects linear regression was used to examine change in BMI slope (kg/m^2^ per month) pre-pandemic to pandemic using FNPA summary and subscales scores as predictors and adjusting for confounding factors.

**Results:**

The analyses included 6,746 children (males: 51.7%, non-Hispanic white: 86.6%, overweight:14.8%, obesity:10.3%, severe obesity: 3.9%; mean(SD) age: 5.7(2.8) years). The rate of BMI change in BMI was greatest from early pandemic compared to pre-pandemic for children in lowest versus highest tertiles of FNPA summary score (0.079 vs. 0.044 kg/m^2^), FNPA-Eating (0.068 vs. 0.049 kg/m^2^), and FNPA-Activity (0.078 vs. 0.052 kg/m^2^). FNPA summary score was significantly associated with change in BMI from the pre-pandemic to early pandemic period (*p* = 0.014), but not associated with change in BMI during the later pandemic period.

**Conclusions:**

This study provides additional insight into the changes in the rate of BMI change observed among children and adolescents in the United States during the COVID-19 pandemic. The FNPA provides ample opportunity to continue our exploration of the negative impact of the COVID-19 pandemic on the longitudinal growth patterns among children and adolescents.

**Supplementary Information:**

The online version contains supplementary material available at 10.1186/s12966-024-01634-2.

## Background

The COVID-19 Pandemic has had markedly negative effects on the longitudinal trends in body mass index (BMI) for children and adolescents in the United States. Within the first year of the pandemic, not only did the rate of BMI significantly increase for children between 2 and 19 years of age, but so too did the prevalence of obesity and severe obesity among this age group [1–3]. Although children in nearly all BMI categories experienced increases in their rate of BMI change, individuals with overweight, obesity, and severe obesity had rates that nearly doubled compared to pre-pandemic [1]. Recent studies have expanded their analyses to compare rates of BMI change during the early pandemic to those as the pandemic progressed [4]. Despite the attenuation in the rate of BMI change during the second half of the pandemic, prevalence of overweight, obesity, and severe obesity among 2–19-year-olds remains high.

Factors contributing to weight gain and increases in BMI among children during the pandemic can be attributed to the ripple effects of the national lockdowns and school closures. Although we generally understand that home environments and behaviors may have shifted to negatively impact the longitudinal growth of children during the pandemic, we know very little about how previously established home environments or behaviors may have operated to protect children from or enhance the increase in BMI observed in the US during the COVID-19 pandemic.

### Objectives

We hypothesized that the same factors key in preventing weight gain in this age group (e.g., consuming a nutrient dense diet, limiting screen time, engaging in moderate to vigorous physical activity) [5] would also be beneficial in buffering the negative impact of the factors exacerbated by the pandemic. As such, our objectives were to evaluate associations between family/home environment and behavioral factors and change in BMI across the pandemic in a longitudinal cohort of children aged 2–17 assessed during annual well-child visits conducted at the Geisinger Health System. Geisinger is a large, integrated health system serving patients in Pennsylvania, USA. Geisinger’s service area covers predominantly rural areas in central and northeast Pennsylvania, and the primary care population represents the region’s general population in terms of age and sex [6].

## Methods

### Study design

This study utilized a retrospective cohort design. Eligible patients were identified via electronic health records (EHR) from Geisinger Health System.

### Setting

Participant data used for this study were those collected during annual well-child visits conducted within the Geisinger Health System in Pennsylvania (USA). Geisinger is a large, integrated health system serving patients in over 40 counties in Pennsylvania. Geisinger’s service area covers predominantly rural areas in central and northeast Pennsylvania, USA. The baseline period is defined as January 1, 2018 through February 29, 2020. Two follow-up periods are the early pandemic period defined as June 1, 2020-December 31, 2020 and the later pandemic period defined as January 1, 2021-December 31, 2021.

### Participants

Eligibility criteria included youth 2–17 years old that received well-child visits at Geisinger prior to and during the pandemic. Participants were selected if their electronic health record contained two or more BMI measurements before the COVID-19 pandemic (with at least one measurement occurring during the year immediately preceding the pandemic: March 1, 2019–February 29, 2020). Furthermore, participants also must have had one or more BMI measurements after the initial 3 months of the pandemic (‘Early Pandemic’); and one or more BMI measurements in the second year of the pandemic (‘Later Pandemic’). Lastly, participants (parents as proxy) must also have had a completed the Family Nutrition and Physical Activity (FNPA) screening tool at a well-child visit in the two years prior to start of COVID-19 pandemic.

### Variables

The exposure variable is Family Nutrition and Physical Activity (FNPA) screening tool completion, and the primary outcome variable is BMI. Other variables of interest included participant age, sex, race/ethnicity, use of public insurance, and COVID-19 Stringency Index [7]. Home obesogenic environments were assessed using the established FNPA tool which has been embedded within the Geisinger system as a standard patient-reported outcome (PRO) measure during well-child visits. The FNPA was developed and validated to assess parenting practices, child behaviors, and home environment characteristics that may predispose children to obesity [8, 9, 11]. When delivered as part of standard well-child visits, FNPA results enable providers to quickly assess a child’s risk and provide personalized preventive counseling. The FNPA risk assessment is a 20-question PRO measure completed by the parent or guardian routinely collected at well child visits [9]. The FNPA summary score (FNPA score) represents the total of all 20 items but 10 items comprise an FNPA-Eating subscale and 10 items comprise an FNPA-Activity subscale [9]. The FNPA-Eating subscale captures 5 constructs with 2 questions each on family meals, family eating practices, food choices, beverage choices, and restriction and reward. Similarly, the FNPA-Activity subscale captures 5 constructs with 2 questions each on screen time, healthy home environment, family physical activity, child physical activity, and sleep routine. The robust nature of the FNPA tool provides a way to explain factors that may have contributed to weight gain during the pandemic.

### Data source/measurement

Participant age, sex, race/ethnicity, FNPA, BMI, and insurance were extracted directly from the EHR. BMI (kg/m^2^) was calculated using height and weight measures collected by clinical staff at the well-child visit and then categorized using CDC sex-specific BMI-for-age percentiles: underweight (< 5th percentile), healthy weight (≥ 5th to < 85th overweight (≥ 85th < 95th obesity (≥ 95th percentile < 120% of the 95th), and severe (≥ 120% 95th) [10]. Prior to evaluating study inclusion criteria, the height, weight, and calculated BMI values were cleaned to remove extreme values, typically the result of data entry errors including BMI < 5 kg/m^2^, BMI > 80 kg/m^2^, BMIz<-4, BMIz > 5, height < 30 cm, and height > 213 cm. The FNPA total scores and subscale scores were computed using the standard scoring methods by summing scores for the full tool, and summing the items contained within FNPA-Eating and FNPA-Activity, respectively [8, 9, 11]. Use of public insurance was a variable informed by the EHR and coded as ever = Yes versus never = No.

### Bias

Select**i**on bias was minimized by restricting the number of inclusion and exclusion criteria to achieve a sample that is representative of the general population in the region, focusing the evaluation of outcomes to the exposed group only, and adjusting for participant characteristics that may explain the outcome.

### Study size

Study size was determined by convenience among a retrospective observational cohort that met inclusion criteria. The study size was sufficiently large as drawn from annual well-child visits where exposure to a treatment (FNPA) and outcomes (anthropometric measures) are documented, per standard care.

### Statistical methods

The primary statistical model was modeled after Pierce et al., [4] such that linear mixed-effects regression models were used to measure the average change in monthly BMI from pre-to during the COVID-19 pandemic. The dependent variable in the model was all BMI measures and the models included a random effect for individual level heterogeneity. Independent variables within the model included linear time, indicator for whether the BMI value was before, during the early pandemic, or during the late pandemic, interaction term between linear time and the two pandemic indicators. Potential sources of bias and confounding were considered in these models by including baseline BMI, sex, age, race/ethnicity, and use of public insurance as a proxy indicator of lower socioeconomic status.

### FNPA model

These models were adapted to test for whether the change in BMI from pre to during the COVID-19 pandemic were associated with FNPA score (using tertiles: lower 1/3 of data, middle 1/3 of data, and highest 1/3 of data). These models included additional terms for baseline FNPA score, interaction of linear time with FNPA tertile, interaction of pandemic indicator with FNPA tertile, and the three-way interaction of linear time, pandemic indicator, and FNPA tertile. Additionally, subscales of the FNPA-Eating and FNPA-Activity were evaluated. Models were run for the overall population and after stratifying by sex. Due to the patient selection process, the data were complete and absent of missing data and loss to follow-up. SAS Viya was used for the regression analysis. P-values < 0.05 were considered significant.

## Results

### Overall study population

A total of 85,214 Geisinger Family Practice or Pediatric patients met baseline BMI inclusion criteria of which 53,987 had a BMI in the Early Pandemic period. Of these, there were a total of 40,628 patients that met Late Pandemic BMI criterion of which 6,746 had a baseline completed FNPA. As compared to those without a completed FNPA (*n* = 33,882), those with the completed FNPA were more likely to be younger at pandemic onset (7.5 years versus 10.9 years, *p* < 0.0001) and non-Hispanic white race (86.6% versus 83.6%, *p* < 0.0001) but were not different for sex (*p* = 0.603) and BMIz (*p* = 0.851).

The longitudinal cohort of 6,746 persons had a total of 72,176 BMI measurements collected from January 1, 2018, through December 31, 2021, including 46,224 pre-COVID; 11,692 during Early Pandemic (COVID Stringency index mean = 66.5, SD = 11.2); and 14,260 during Later Pandemic (COVID Stringency Index mean = 54.8, SD = 6.5) [7]. At baseline, the majority of participants were male (51.7%), between 2 and 5 years of age (57.3%), and identified as non-Hispanic white (86.6%) (Table 1). The proportion of children with overweight, obesity, and severe obesity were 14.3%, 10.8%, and 3.9% respectively.

**Table 1:** Baseline characteristics of the Longitudinal Cohort prior to the COVID-19 pandemic

Baseline characteristics		% (*n*)
Age at initial BMI measure	2–5 years	57.3% (*n* = 3864)
	6–11 years	42.7% (*n* = 2882)
	Mean (SD)	5.7 (2.8)
Age at pandemic onset	2–5 years	38.7% (*n* = 2607)
	6–11 years	53.3% (*n* = 3597)
	12–17 years	8.0% (*n* = 542)
	Mean (SD)	7.5 (2.9)
Sex	Male	51.7% (*n* = 3488)
	Female	48.3% (*n* = 3258)
Race/ethnicity	Non-Hispanic white	86.6% (*n* = 5842)
	Non-Hispanic black	4.2% (*n* = 285)
	Hispanic	7.6% (*n* = 515)
	Asian	1.1% (*n* = 76)
	Other/Unknown	0.4% (*n* = 28)
Initial BMI category	< 5th %tile	4.5% (*n* = 300)
	5–49%tile	31.4% (*n* = 2120)
	50–84%tile	35.1% (*n* = 2370)
	85–94%tile	14.3% (*n* = 966)
	95%-<120% BMI95	10.8% (*n* = 726)
	>=120%BMI95	3.9% (*n* = 264)
FNPA summary score	Mean (SD)	64.9 (6.6)
Insurance type	Public	29.5% (*n* = 1988)
	Private	70.5% (*n* = 4758)

### FNPA model results

We observed accelerated rates of BMI change across tertiles within FNPA score and the FNPA subscales (Table 2). Monthly change in BMI was greatest from early pandemic compared to pre-pandemic for children in lowest versus highest tertiles of FNPA summary score (0.079 vs. 0.044 kg/m^2^), FNPA-Eating (0.068 vs. 0.049 kg/m^2^), and FNPA-Activity (0.078 vs. 0.052 kg/m^2^). Slightly larger differences were observed among males but not females in subgroup analyses (data not shown; Additional file 1). FNPA score was significantly associated with relative change in BMI from the pre-pandemic to early pandemic period (*p* = 0.014), but not associated with relative change in BMI during the later pandemic period. Children with FNPA summary scores in Tertile 1 exhibited a 156% increase in BMI rate compared to a 141% increase for those in Tertile 3. This result held among males but not females and may have been driven by more favorable FNPA-Activity scores (Additional file 1). Findings among females were not statistically different except among the Later Pandemic period when comparing FNPA-Eating subscale tertiles (Additional file 1). We also observed an attenuation in the rate of BMI change during the later pandemic from the accelerated rates observed in early pandemic, with rates returning close to pre-pandemic rates across tertiles. These findings were consistent among female/male subgroup analyses.

**Table 2:** Model comparing change in BMI (kg/m^2^ change per month) from pre to during COVID-19 pandemic periods between varying levels of FNPA summary scores, eating, and activity subscales

	Pre- Pandemic	Early Pandemic	Difference in Early vs. Pre	*p*-value1	Late Pandemic	Difference in Late vs. Pre	*p*-value2
**FNPA Summary Score**							
Tertile 1: 38–62 (*n* = 2221)	0.050 (0.0016)	0.128 (0.0092)	0.079 (0.0093)	Ref	0.054 (0.0064)	0.004 (0.0067)	Ref
Tertile 2: 63–68 (*n* = 2405)	0.038 (0.0015)	0.097 (0.0095)	0.059 (0.0096)	0.153	0.041 (0.0064)	0.003 (0.0067)	0.944
Tertile 3: 69–80 (*n* = 2120)	0.031 (0.0017)	0.075 (0.0104)	0.044 (0.0105)	**0.014**	0.046 (0.0074)	0.015 (0.0076)	0.266
**FNPA-Eating**							
Tertile 1: 14–30 (*n* = 1736)	0.049 (0.0018)	0.117 (0.0106)	0.068 (0.0107)	Ref	0.052 (0.0074)	0.003 (0.0076)	Ref
Tertile 2: 31–34 (*n* = 3306)	0.038 (0.0013)	0.103 (0.0080)	0.064 (0.0081)	0.769	0.048 (0.0056)	0.010 (0.0099)	0.441
Tertile 3: 35–40 (*n* = 1704)	0.033 (0.0019)	0.082 (0.0117)	0.049 (0.0118)	0.228	0.034 (0.0083)	0.001 (0.0085)	0.909
**FNPA-Activity**							
Tertile 1: 14–30 (*n* = 1948)	0.053 (0.0017)	0.132 (0.0098)	0.078 (0.0099)	Ref	0.055 (0.0069)	0.002 (0.0071)	Ref
Tertile 2: 31–34 (*n* = 2354)	0.034 (0.0016)	0.088 (0.0095)	0.054 (0.0097)	0.078	0.036 (0.0066)	0.002 (0.0068)	0.989
Tertile 3: 35–40 (*n* = 2444)	0.034 (0.0015)	0.086 (0.0097)	0.052 (0.0098)	0.058	0.048 (0.0068)	0.014 (0.0070)	0.225

Values are mean change in BMI per month (SE) adjusted for baseline BMI, sex, race/ethnicity, age, and public insurance

p-value1 = comparison of difference in BMI change from pre-pandemic and early pandemic (comparing Tertile 2 and Tertile 3 versus Tertile 1)

p-value2 = comparison of difference in BMI change from pre-pandemic and late pandemic (comparing Tertile 2 and Tertile 3 versus Tertile 1)

## Discussion

The COVID-19 pandemic presented numerous obstacles and set-backs relative to the health and well-being of children and adolescents in the United States. With this analysis we sought to understand whether change in BMI from pre-COVID to two time points during the COVID-19 pandemic was associated with pre-established home environments and behaviors, measured by the FNPA tool. A high FNPA score generally indicates low obesity risk while a low score generally indicates high obesity risk. In terms of our key result, we found that higher FNPA summary score in the pre pandemic period was associated with less BMI gain during the early pandemic compared to children with lower FNPA scores. These findings were expected, as children with established healthy home environments and behaviors prior to the onset of the pandemic likely maintained some protection against rapid BMI gain once the pandemic began. However, all children, regardless of FNPA score, had rapid relative BMI increases in early pandemic compared to pre-pandemic period, despite higher scores demonstrating less of an increase. Others have described the disruptions to children’s lifestyles that contributed to rapid BMI increases during the early pandemic. By the spring of 2020, 77% of public schools in the US reported switching to a distance-learning format from in-person [12]. A European meta-analysis concluded that the decline in physical activity recorded during the pandemic was highest during period of school closures [13]. Not only did the pandemic increase ‘out of school time,’ a significant contributor to childhood weight gain observed during the summer months [14], but the pandemic also exacerbated risk factors for weight gain by creating a more obesogenic environment at home. Reports demonstrated that the prevalence of food insecurity increased [15], screen time increased and physical activity decreased [16], and children experienced an increase in anxiety and depressive symptoms [17].

Notably, we did not observe an association between FNPA score with changes in the rate of BMI from pre-pandemic to later-pandemic. This trend has been similarly observed in the literature. Pierce et al., analyzed a longitudinal cohort of 241,600 children aged 2–19 to examine differences in rates of change in BMI, weight, and obesity prevalence across a pre-pandemic and two pandemic time periods. Compared to the accelerated rates of BMI observed during the early pandemic period (2020), the later pandemic period (2021) displayed diminished but still positive rates of BMI change [4]. Children in summary score tertiles 1 and 2 exhibited similar attenuation of BMI rate, such that rates observed in the later pandemic closely resembled those from pre-pandemic. Children in summary score tertile 3, however, displayed rates that remained higher than pre-pandemic rates. The conditions of the later pandemic were different from that of the early pandemic, most notably with a return to in-person learning and fewer school closings. Where the early pandemic was marked by substantial ‘out of school time’ with accelerated BMI, resembling trends often associated with summer break [14, 18], the later pandemic saw the restoration of not only reliable sources of physical activity for children, but also for nutritious meals [19]. Longitudinal analyses in COVID-19 related health behavior changes among children have revealed similar observations with pandemic-induced changes in sleep, dietary habits, and physical activity returning closely to pre-pandemic behaviors by 2021 [20].

We chose to evaluate subscales of the FNPA summary score as FNPA-Eating and FNPA-Activity to delineate the concepts within the FNPA that most closely reflected family or child behaviors related to diet or food versus those related to physical activity. FNPA-Eating and FNPA-Activity still represent all questions of the FNPA. Previous studies have evaluated other subscales within the FNPA including just those related to physical activity or sleep [21]. Within the FNPA-Eating results we see that children with scores in Tertile 3 have later-pandemic BMI rates that return very close to pre-pandemic rates, while those with Tertile 3 scores within FNPA-Activity have later-pandemic BMI rates that remain higher than pre-pandemic. Reports have indicated parents perceived their children’s intensity and duration of physical activity declined during the pandemic [20], but there is limited objective data for such changes. Our findings suggest sex-effects factor into the association between physical activity and BMI during the pandemic. Among males only, BMI gains in the early pandemic period were significantly lower among those with FNPA scores in Tertile 3 versus Tertile 1. Consistency of health behaviors and routines were impacted at variable stages and degrees of the pandemic; the endurance of FNPA assessment of home environments and practices during the pandemic may not permeate across multiple timeframes.

There is general consensus that effective obesity prevention should target: (1) poor diet (e.g., consumption of sugar-sweetened beverages and energy-dense foods); (2) low levels of physical activity; (3) short sleep duration; (4) sedentary behaviors (e.g., high media use); and (5) parenting practices [22–25]. The FNPA measure and risk assessment addresses all 5 behaviors and is a valid clinical tool to identify risk factors associated with obesity [8, 9, 11] among children [26] and related chronic disease indicators (adiposity measures, severity of obesity, cardiovascular disease risk, and glucose intolerance) [27–29]. What’s more, the FNPA risk assessment offers time efficiencies to clinicians as parents self-assess risk to allow the primary care provider to focus discussion on relevant, family-centered issues [26, 30]. Given the utility of the tool, we sought to understand whether FNPA surveys completed prior to the pandemic might provide new insight into the change in longitudinal trends in BMI observed among children and adolescents. We observed short-term protective effects among those with higher FNPA scores versus lower scores overall and among males. Clincially, there may be utility in annually collecting the FNPA tool and using current scores to inform and provide timely, relevant preventive counseling. Use of FNPA as a PRO measure during well-child visits at one institution could advance population health by preventing child obesity FNPA, and wider adoption among many health care systems may advance public health objectives.

We acknowledge that this study possesses a few limitations. First the sample size included was limited by requiring an additional BMI measure captured within the later pandemic window of 2021. The population included in this analysis, while representative of Central Pennsylvania and the Geisinger Service Region, is not representative of the whole United States. Lastly, the FNPA scores (total summary score and Eating and Activity subscales) used here represent pre-pandemic values as opposed to change in scores across pandemic. However, FNPA summary score is significantly inversely associated with obesity risk and anchoring this study with scores prior to the start of the pandemic was an intentional choice relative to preexisting risk. We chose to evaluate the subscales of Eating and Activity in addition to FNPA summary score, however, these subscales are the result of condensing several questions into one subscale score. In the future we intend to investigate the individual contribution of items within each subscale to the associations observed, particularly in the early pandemic. Additionally, we hope to evaluate patients with FNPA surveys completed across the pandemic and examine change in FNPA scores across pre- and during the pandemic in association with changes in BMI.

## Conclusions

This study provides additional insight into the changes in the rate of BMI change observed among children and adolescents in the United States during the COVID-19 pandemic. With the collection of the FNPA as a clinical PRO measure, we assessed how preexisting measures of family home environment and behaviors associated with the rate of change in BMI among 2–17 year olds across the pandemic. The FNPA provides ample opportunity to continue our exploration of the negative impact of the COVID-19 pandemic on the longitudinal growth patterns among children and adolescents.

### Electronic supplementary material

Below is the link to the electronic supplementary material.

Supplementary Material 1

Supplementary Material 2

Supplementary Material 3

## Data Availability

The datasets generated and/or analyzed during the current study are not publicly available due to being protected electronic health record data.

## Abbreviations

PRO: Patient-reported outcome
BMI: Body mass index
IRB: Institutional Review Board
FNPA: Family nutrition and physical activity
EHR: Electronic health record
