# Supplementary material for: Home environment factors associated with child BMI changes during COVID-19 pandemic
Source: Int J Behav Nutr Phys Act. 2024 Aug 2;21:84. doi: 10.1186/s12966-024-01634-2 (PMC11295326; doi:10.1186/s12966-024-01634-2)
Supplement: Supplementary file 1 — Supplementary Material 1 [file 12966_2024_1634_MOESM1_ESM.docx]

**Additional file 1**. Model comparing change in BMI (kg/m^2^ change per month) from pre to during COVID-19 pandemic periods between varying levels of FNPA summary scores, eating, and activity subscales among females only.

|  | Pre- Pandemic | Early Pandemic | Difference in Early vs Pre | p-value1 | Late Pandemic | Difference in Late vs Pre | p-value2 |
| --- | --- | --- | --- | --- | --- | --- | --- |
| **FNPA Summary Score** |  |  |  |  |  |  |  |
| Tertile 1: 38-62 (n=1062) | 0.054 (0.0022) | 0.134 (0.0130) | 0.080 (0.0144) | Ref | 0.057 (0.0091) | 0.003 (0.0101) | Ref |
| Tertile 2: 63-68 (n=1123) | 0.044 (0.0022) | 0.097 (0.0139) | 0.053 (0.0139) | 0.166 | 0.060 (0.0094) | 0.016 (0.0096) | 0.3678 |
| Tertile 3: 69-80 (n=1073) | 0.024 (0.0023) | 0.089 (0.0146) | 0.065 (0.0134) | 0.470 | 0.037 (0.0103) | 0.013 (0.0096) | 0.466 |
| **FNPA-Eating** |  |  |  |  |  |  |  |
| Tertile 1: 14-30 (n=803) | 0.057 (0.0026) | 0.129 (0.0152) | 0.072 (0.0158) | Ref | 0.043 (0.0103) | -0.014 (0.0109) | Ref |
| Tertile 2: 31-34 (n=1579) | 0.039 (0.0019) | 0.106 (0.0116) | 0.067 (0.0121) | 0.815 | 0.064 (0.0080) | 0.025 (0.0085) | **0.0039** |
| Tertile 3: 35-40 (n=876) | 0.029 (0.0026) | 0.091 (0.0161) | 0.062 (0.0146) | 0.643 | 0.035 (0.0115) | 0.005 (0.0106) | 0.228 |
| **FNPA-Activity** |  |  |  |  |  |  |  |
| Tertile 1: 14-30 (n=948) | 0.060 (0.0026) | 0.129 (0.0137) | 0.069 (0.0154) | Ref | 0.070 (0.0096) | 0.010 (0.0110) | Ref |
| Tertile 2: 31-34 (n=1106) | 0.039 (0.0022) | 0.101 (0.0137) | 0.063 (0.0138) | 0.741 | 0.037 (0.0094) | -0.002 (0.0096) | 0.397 |
| Tertile 3: 35-40 (n=1204) | 0.029 (0.0022) | 0.094 (0.0139) | 0.065 (0.0127) | 0.817 | 0.049 (0.0096) | 0.021 (0.0089) | 0.455 |

Values are mean change in BMI per month (SE) adjusted for baseline BMI, sex, race/ethnicity, age, and public insurance.

p-value1=comparison of difference in BMI change from pre-pandemic and early pandemic (comparing Tertile 2 and Tertile 3 versus Tertile 1)

p-value2=comparison of difference in BMI change from pre-pandemic and late pandemic (comparing Tertile 2 and Tertile 3 versus Tertile 1)

Model comparing change in BMI (kg/m^2^ change per month) from pre to during COVID-19 pandemic periods between varying levels of FNPA summary scores, eating, and activity subscales among males only.

|  | Pre- Pandemic | Early Pandemic | Difference in Early vs Pre | p-value1 | Late Pandemic | Difference in Late vs Pre | p-value2 |
| --- | --- | --- | --- | --- | --- | --- | --- |
| **FNPA Summary Score** |  |  |  |  |  |  |  |
| Tertile 1: 38-62 (n=1159) | 0.046 (0.0025) | 0.123 (0.0130) | 0.076 (0.0151) | Ref | 0.050 (0.0092) | 0.004 (0.0109) | Ref |
| Tertile 2: 63-68 (n=1282) | 0.032 (0.0021) | 0.096 (0.0130) | 0.064 (0.0124) | 0.501 | 0.024 (0.0091) | -0.008 (0.0086) | 0.367 |
| Tertile 3: 69-80 (n=1047) | 0.038 (0.0024) | 0.061 (0.0148) | 0.022 (0.0132) | **0.0065** | 0.055 (0.0105) | 0.017 (0.0095) | 0.393 |
| **FNPA-Eating** |  |  |  |  |  |  |  |
| Tertile 1: 14-30 (n=933) | 0.042 (0.0025) | 0.108 (0.0147) | 0.065 (0.0159) | Ref | 0.060 (0.0105) | 0.018 (0.0115) | Ref |
| Tertile 2: 31-34 (n=1727) | 0.038 (0.0181) | 0.099 (0.0111) | 0.061 (0.0114) | 0.807 | 0.033 (0.0078) | -0.004 (0.0081) | 0.100 |
| Tertile 3: 35-40 (n=828) | 0.036 (0.0027) | 0.072 (0.0168) | 0.037 (0.0152) | 0.199 | 0.033 (0.0118) | -0.003 (0.0108) | 0.196 |
| **FNPA-Activity** |  |  |  |  |  |  |  |
| Tertile 1: 14-30 (n=1000) | 0.047 (0.0027) | 0.133 (0.0139) | 0.086 (0.0164) | Ref | 0.041 (0.0098) | -0.006 (0.0118) | Ref |
| Tertile 2: 31-34 (n=1248) | 0.031 (0.0022) | 0.077 (0.0132) | 0.047 (0.0125) | **0.043** | 0.036 (0.0092) | 0.005 (0.0088) | 0.400 |
| Tertile 3: 35-40 (n=1240) | 0.039 (0.0021) | 0.080 (0.0135) | 0.041 (0.0124) | **0.020** | 0.047 (0.0096) | 0.0079 (0.0089) | 0.315 |

Values are mean change in BMI per month (SE) adjusted for baseline BMI, sex, race/ethnicity, age, and public insurance.

p-value1=comparison of difference in BMI change from pre-pandemic and early pandemic (comparing Tertile 2 and Tertile 3 versus Tertile 1)

p-value2=comparison of difference in BMI change from pre-pandemic and late pandemic (comparing Tertile 2 and Tertile 3 versus Tertile 1)
