# Supplementary material for: Home environment factors associated with child BMI changes during COVID-19 pandemic
Source: Int J Behav Nutr Phys Act. 2024 Aug 2;21:84. doi: 10.1186/s12966-024-01634-2 (PMC11295326; doi:10.1186/s12966-024-01634-2)
Supplement: Supplementary file 3 — Supplementary Material 3 [file 12966_2024_1634_MOESM3_ESM.pdf]

The Family Nutrition & Physical Activity Screening Tool!

Instructions: For each question, select the answer category that best fits your child or your family. It is important to indicate the most common or typical pattern for your family, and not what you would like to happen.

**Family Meals**	Current Build in DatStat/Epic	Never/ Almost Never	Sometimes	Often	Very Often/ Always
1. How often does your child eat breakfast, either at home or at school?	My child eats breakfast	1	2	3	4
2. How often does your child eat at least one meal a day with at least one other family member?	Our family eats meals together	1	2	3	4
**Family Eating Practices**		Never/ Almost Never	Sometimes	Often	Very Often/ Always
3. How often does your child eat while watching TV? [Includes meals or snacks]	Our family eats while watching TV	1	2	3	4
4. How often does your family eat "fast food?"	Our family eats fast food	1	2	3	4
**Food Choices**		Never/ Almost Never	Sometimes	Often	Very Often/ Always
5. How often does your family use packaged "ready--to--eat" foods? [Includes purchased frozen or on--the--shelf entrees, often designed to be microwaved]	Our family uses microwave or ready to eat foods	1	2	3	4
6. How often does your child eat fruits and vegetables at meals or snacks? [Not including juice]	My child eats fruits and vegetables at meals or snacks	1	2	3	4
**Beverage Choices**		Never/ Almost Never	Sometimes	Often	Very Often/ Always
7. How often does your child drink soda pop or sweetened beverages? [Includes regular or diet soda pop, Kool--Aid, Sunny--D, Capri Sun, fruit or vegetable juice, caffeinated energy drinks (Monster/Red Bull), Powerade/Gatorade, etc.]	My child drinks soda pop or sugar drinks	1	2	3	4

8. How often does your child drink low--fat milk for meals or snacks? [Includes 1% or skim dairy, flavored, soy, almond, etc.]	My child drinks low fat milk at meals or snacks	1	2	3	4
**Restriction/Reward**		Never/ Almost Never	Sometimes	Often	Very Often/ Always
9. How often does your family monitor the amount of candy, chips, and cookies your child eats?	Our family limits eating of chips, cookies, and candy	1	2	3	4
10. How often does your family use candy, ice cream or other foods as a reward for good behavior?	Our family uses candy as a reward for good behavior	1	2	3	4

**Screen Time**		Never/ Almost Never	Sometimes	Often	Very Often/ Always
11. How often does your child have less than 2 hours of "screen time" in a day? [Includes TV, computer, game system, or any mobile device with visual screens]	My child spends less than 2 hours on TV/games/computer per day	1	2	3	4
12. How often does your family monitor the amount of "screen time" your child has?	Our family limits the amount of TV our child watches	1	2	3	4
**Healthy Environment**		Never/ Almost Never	Sometimes	Often	Very Often/ Always
13. How often does your child engage in screen time in his/her bedroom?	Our family allows our child to watch TV in their bedroom	1	2	3	4
14. How often does your family provide opportunities for physical activity?	Our family provides opportunities for physical activity	1	2	3	4
**Family Activity**		Never/ Almost Never	Sometimes	Often	Very Often/ Always
15. How often does your family encourage your child to be physically active?	Our family encourages our child to be active every day	1	2	3	4
16. How often does your child do physical activities with at least one other family	Our family finds ways to be physically active together	1	2	3	4

**Child Activity**		Never/ Almost Never	Sometimes	Often	Very Often/ Always
17. How often does your child do something physically active when he/she has free time?	My child does physical activity during his/her free time	1	2	3	4
18. How often does your child participate in organized sports or physical activities with a coach or leader?	My child is enrolled in sports or activities with a coach or leader	1	2	3	4
**Family Schedule/Sleep Routine**		Never/ Almost Never	Sometimes	Often	Very Often/ Always
19. How often does your child follow a regular routine for your child's bedtime?	Our family has a daily routine for our child's bedtime	1	2	3	4
20. How often does your child get enough sleep at night?	My child gets 9 hours of sleep a night	1	2	3	4

The FNPA tool was developed at Iowa State University by Michelle Ihmels and Greg Welk (gwelk@iastate.edu) in partnership with the American Dietetics Association

Scoring the FNPA

A number of the items on the FNPA are reverse scored with “Very Often/Always” being the less desirable options, so care should be taken when calculating a total score.

Items to be Reverse Scored (Construct):

3 Family Eating Practices

4 Family Eating Practices

5 Food Choices

7 Beverage Choices

10 Restriction/Reward

13 Healthy Environment

No cut points or threshold have been established for determining Healthy vs Unhealthy home environments. Researchers and practitioners should use their own discretion when interpreting scores on the FNPA.

Additional resources can be found at myfnpa.org and eatright.org.
